# Cross-sectional and longitudinal neural predictors of physical activity and sedentary behaviour from a 6-month randomized controlled trial

**DOI:** 10.1038/s41598-023-48715-z

**Published:** 2024-01-09

**Authors:** Ryan Stanley Falck, Chun Liang Hsu, John R. Best, Narlon Cassio Boa Sorte Silva, Peter A. Hall, Linda C. Li, Teresa Liu-Ambrose

**Affiliations:** 1 Djavad Mowafaghian Centre for Brain Health, Vancouver Coastal Health Research Institute, University of British Columbia, Vancouver, BC Canada; 2 Centre for Aging SMART at Vancouver Coastal Health, Vancouver Coastal Health Research Institute, University of British Columbia, Vancouver, BC Canada; 3 Department of Rehabilitation Sciences, The Hong Kong Polytechnic University, Hung Hom, Hong Kong; 4 Gerontology Research Centre, Simon Fraser University, Vancouver, BC Canada; 5 School of Kinesiology, The University of Waterloo, Waterloo, ON Canada; 6 Department of Physical Therapy, Faculty of Medicine, The University of British Columbia, Vancouver, BC Canada; 7 Aging, Mobility, and Cognitive Neuroscience Lab, Department of Physical Therapy, Vancouver Coastal Health Research Institute, Faculty of Medicine, University of British Columbia, 212–177 Wesbrook Mall, Vancouver, BC V6T 1Z3 Canada

**Keywords:** Psychology, Human behaviour, Neuroscience, Medical research, Risk factors

## Abstract

A sedentary lifestyle offers immediate gratification, but at the expense of long-term health. It is thus critical to understand how the brain evaluates immediate rewards and long-term health effects in the context of deciding whether to engage in moderate-to-vigorous physical activity (MVPA) or sedentary behaviour (SB). In this secondary analysis of a 6-month randomized controlled trial to increase MVPA and reduce SB among community-dwelling adults, we explored how neural activity during an executive control task was associated with MVPA and SB levels. At baseline, a subset of participants (n = 26/61) underwent task-based functional magnetic resonance imaging (fMRI) to examine neural activity underlying executive control using the Now/Later task. MVPA and SB were measured objectively using the Sensewear Mini at baseline, and 2, 4, and 6 months follow-up. We then examined the associations of baseline neural activation underlying executive control with: (1) baseline MVPA or SB; and (2) changes in MVPA and SB over 6 months. Our results determined that there is a complex neurocognitive system associated with MVPA levels, while SB appears to lack any neurocognitive control. In other words, MVPA appears to require neurocognitive effort, while SB may be the default behavioural pattern in adults.

## Introduction

Regular moderate-to-vigorous physical activity (MVPA), bodily movement which increases energy expenditure ≥ 3.0 metabolic equivalents (METs)^1^, substantially reduces morbidity and mortality^2^. By comparison, higher amounts of sedentary behaviour (SB)—behaviour which expends ≤ 1.5 METs and is performed while seated or lying down^3^—is linked to chronic conditions and increased mortality risk^4^. Guidelines broadly suggest that adults should: (1) engage in at least 150 min/week of MVPA; and (2) limit SB as much as possible^5,6^.

Less than 10% of U.S. adults met MVPA guidelines in 2006^7^; adherence to MVPA guidelines did not change from 2007 to 2016, while average daily SB increased from 5.7 to 6.4 h/day during this same time span^8^. From 2001 to 2016, total time spent sitting among older adults increased from 5.3 to 6.1 h/day^9^. Predictors of adherence to current MVPA and SB guidelines are likely multi-faceted and due to a combination of individual (e.g., frailty status or physical fitness,) perceptual (e.g., safety and accessibility of MVPA), behavioural, and environmental factors^10^. Current events such as the COVID-19 pandemic and climate change may also contribute to lower MVPA and higher SB levels due to hospitalization, sustained quarantine, and social distancing and acutely hot temperatures which reduce activity levels, respectfully^11,12^. In addition, people with chronic conditions—such as knee osteoarthritis—might also have different barriers and facilitators, such as pain and physical limitations, osteoarthritis related distress, and access to healthcare professionals^13^.

Despite failing to meet guidelines, most adults perceive MVPA as beneficial for their health^14^ and recognize the negative health consequences of too much SB^15^. There are costs (e.g., physical discomfort) and barriers (e.g., time) associated with MVPA^16^. Thus, regular MVPA engagement requires significant self-regulatory capacity (i.e., cognitive control over behaviour in the pursuit of long term goals). Individuals with greater self-regulatory capacity are more successful at implementing MVPA intentions, and are more active and less sedentary because they can override impulses for immediate rewards and shift focus to delayed outcomes^17,18^. In contrast, engagement in SB requires little-to-no self-regulatory capacity, suggesting SB may be the default behavioural pattern^19,20^.

The neurocognitive processes which underly self-regulatory capacity are collectively described as executive control—a set of cognitive processes which serve to enable reflective, “top-down” control of behaviour with important nodes in the prefrontal cortex^21^. MVPA and SB share a bidirectional relationship with executive control performance—whereby greater MVPA and less SB predict better performance, and vice versa^22–24^. However, the association between SB and executive function appears to be substantially weaker than the association between MVPA and executive function^25,26^.

MVPA and SB may also share a bidirectional relationship with the structural neural correlates of executive control. Greater MVPA predicts slower cortical thinning over five years across multiple regions underlying executive control^27^; it is unclear if SB predicts brain structural changes, although SB is cross-sectionally associated with less brain volume in regions associated with executive performance^28^. Conversely, greater brain volume in regions associated with executive control predict MVPA and SB^29,30^. Less is known regarding the relationship between MVPA, SB, and functional neural correlates of executive functions. MVPA is associated with functional neural correlates of executive control^31^, and resting state functional connectivity between regions underlying executive control predicts change in SB^32^. However, no one study has examined and compared functional neural correlates of executive control which predict MVPA and SB and their changes. A better understanding will improve future intervention development and precision medicine approaches^32^.

The purpose of this study was thus to investigate how neural activity during an executive control task is associated with MVPA and SB levels. We hypothesized that adults with greater baseline cortical activation underlying executive control would have: (1) higher baseline MVPA and lower SB; and (2) greater increases in MVPA and reduced SB over 6 months.

## Methods

### Study design

This was a planned neuroimaging sub-study of the *Monitor-OA* study—a 6-month delayed-control design randomized controlled trial (RCT) which examined the efficacy of a technology-enabled counselling intervention to increase MVPA and reduce SB in 61 adults with knee osteoarthritis^33^. The research protocol was approved by the University of British Columbia Clinical Research Ethics Board (Application number: H14-01762), and registered with ClinicalTrials.gov (Registration Number: NCT02315664; Date registered: 12/12/2014). The study occurred between November 1st 2015 and June 1st 2017. All participants provided written informed consent. All methods were performed in accordance with relevant guidelines and regulations.

**Figure 1:**
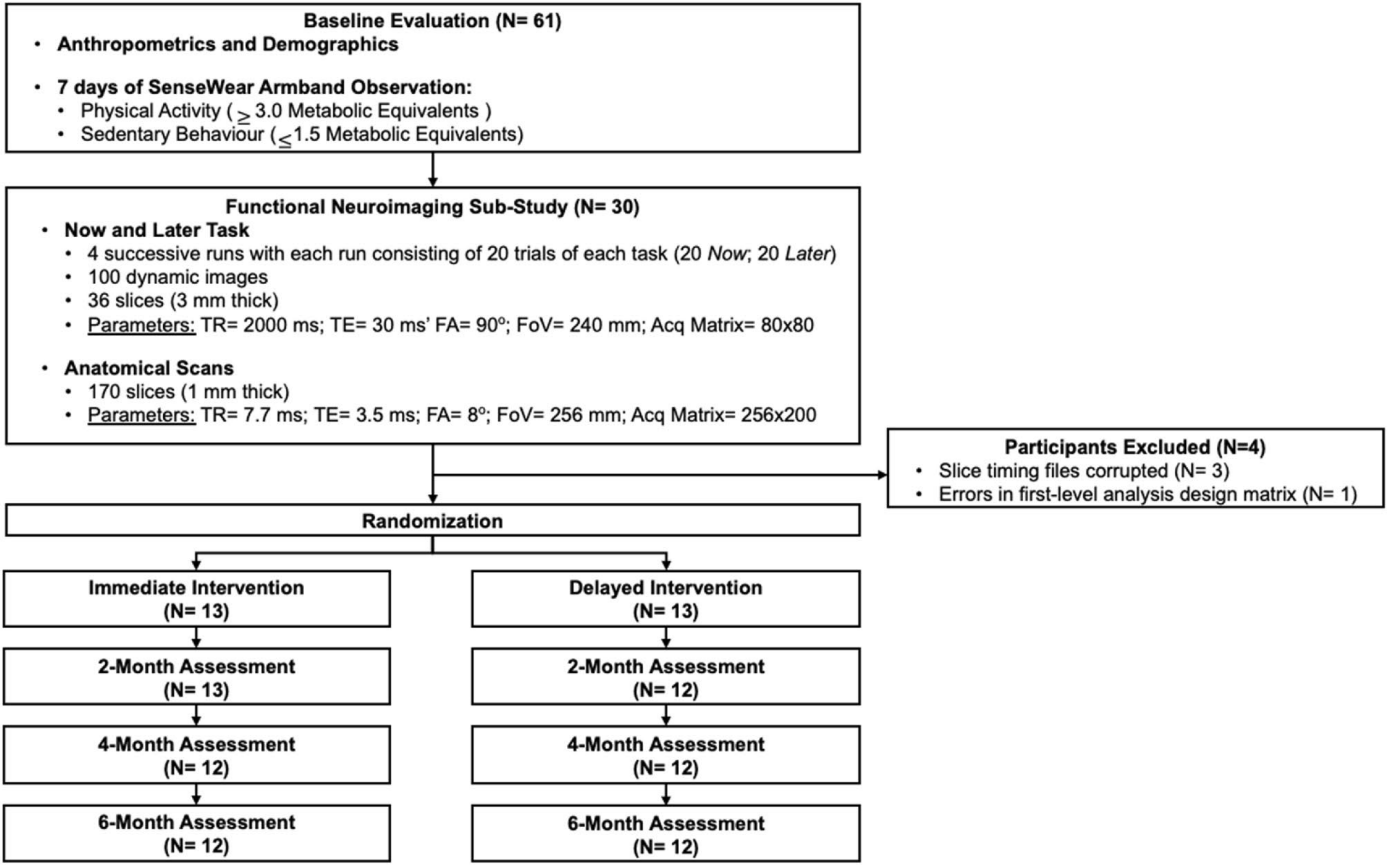
CONSORT diagram.

Figure 1 describes the study design of *Monitor-OA*. At baseline, a sub-sample of 30 right-handed participants were recruited for this sub-study and underwent magnetic resonance imaging (MRI) scanning at baseline after completing all other baseline measurements (i.e., demographics, MVPA, and SB). Participant MVPA and SB were re-assessed at 2 months, 4 months, and 6 months post randomization. Following baseline assessments, all 61 participants were randomly allocated to either an immediate intervention group (I-INT) or a two-month delayed intervention group (D-INT). The I-INT group received the intervention following randomization at baseline to two-month follow-up; D-INT received the intervention from two months to four months. Neither group received the intervention from four months to 6 months. In the primary study, we found that the 2-month intervention significantly increased MVPA and improved quality of life relative to the control following the intervention^33^.

### Participants

We recruited individuals with physician confirmed knee osteoarthritis, or (1) aged 50+ years; and (2) had experienced knee pain or discomfort lasting > 28 days within the last 12 months. Participants were excluded for: (1) living with a diagnosis of inflammatory arthritis, connective tissue diseases, fibromyalgia, or gout; (2) using disease modifying anti-rheumatic drugs or gout medications; (3) planning to or having received total knee arthroplasty; (4) had an acute knee injury or received hyaluronate or steroid injections in the last 6 months; (5) did not have an email address or daily access to a personal computer with internet; (6) a body mass index (BMI) of > 40 kg/m^2^; or (7) using medications that impaired activity tolerance (e.g., beta-blockers). Eligibility criteria were assessed via self-report during screening, except for knee osteoarthritis diagnosis which was ascertained from the participant’s primary care physician. In addition, potential participants completed the Physical Activity Readiness Questionnaire (PAR-Q)^34^. If a potential risk was identified by the PAR-Q, physician confirmation in writing was required to confirm the individual can be physically active without being supervised by a health professional.

### Intervention

All participants received the two-month intervention. Participants in the I-INT group received the intervention from baseline to two months; the D-INT group received the intervention from two months to four months.

Participants first attended a 1.5-h session, where they received: (1) standardized group education about MVPA and SB, (2) a Fitbit^®^ Flex™, and (3) individual activity counselling with a physiotherapist. The education session addressed the benefits of increasing MVPA and reducing SB. The individual counselling component used the Brief Action Planning approach to promote MVPA and reduce SB^35^. The physiotherapist guided participants to identify MVPA goals, develop an action plan, and then identify barriers and solutions. The physiotherapist used the SMART (specific, measurable, attainable, relevant, time-bound) principle during goal setting. Participants were then asked to rate their confidence to executing their plan on a zero to 10 scale, with 10 meaning very confident. The process was repeated until the confidence rating reached 7 or higher out of 10. For SB, the physiotherapist began by asking participants to estimate their time sitting in a normal day and identify ways to break up the sitting time. They then repeated the goal setting and confidence assessment.

Participants were then provided a Fitbit Flex to be worn on the wrist 24 h/day except during water-based activity or when charging; a research assistant educated participants on the use of the device (e.g., proper wear, charging, precautions regarding skin reactions) and its’ features. The Fitbit Flex has evidence of validity and reliability for measuring activity levels, and thus we used the device for monitoring participant adherence to their MVPA goals^36^. Data from the Fitbit Flex were synchronized with Fitbit’s online Dashboard, and could only be viewed by the participants and their study physiotherapist.

During the intervention period, the physiotherapist reviewed participant activity data and progressively modified activity goals during four biweekly phone calls. The physiotherapist used their clinical judgement to help participants modify their MVPA and SB goals using the Brief Action Planning approach^35^. Briefly, the physiotherapist would counsel the participants to make decisions about modifying their goals during bi-weekly phone calls; however, these activity goals were ultimately set by the participant. For example, if the participant set a goal of 100 min/week of MVPA, then this would be their goal. At the end of the intervention, participants kept the Fitbit Flex.

### Measures

#### General health and demographics

General health history and demographics were obtained by questionnaire. Global cognitive function was indexed using the Montreal Cognitive Assessment^37^. Participant knee osteoarthritis symptom severity was indexed using the Knee Injury and Osteoarthritis Outcome Severity (KOOS) score^38^.

#### Objectively-measured MVPA and SB

We measured MVPA and SB at baseline prior to MRI data collection and two months, four months, and 6 months using the SenseWear Mini (Body Media, Pittsburgh, PA, USA), a multimodal device which provides valid and reliable estimates of MVPA and SB^39,40^. The device uses proprietary algorithms (SenseWear professional 7.0 software) to extract daily MVPA and SB estimates by integrating tri-axial accelerometer data, physiological sensor data (i.e., heat flux, galvanic skin responses, skin temperature, and near-body ambient temperature), and personal demographic information (e.g., age, sex, height, and weight). Sleep time is automatically extracted from the data (for more details, see link) using a proprietary algorithm which has evidence of validity and reliability^41^. Off-body wear time is automatically determined when the device is not in contact with the body. Participants were also asked to log when they took off the device, and for how long and what purpose—which was then used for cross-validation if needed. Participant data were extracted at each time point when there was a minimum of 4/7 days of wear time > 20 h, as per the recommendations of Almeida and colleagues^42^. Wear time did not need to be on consecutive days, and we used the mean of all days worn to define MVPA and SB outcomes. Mean wear time for the device was 5.94 days (SD = 0.31), indicating that at least one weekend day was included. At each timepoint, all available participants provided valid data.

### Task-based fMRI data acquisition and processing

At baseline, the 30 MRI sub-study participants had T_1_-weighted structural MRI images obtained using a Philips Achieve 3 T scanner with an eight-channel sensitivity encoding neurovascular coil (SENSE factor = 2.4). High-resolution T_1_-weighted images were obtained according to the following parameters: (1) isotropic spatial resolution of 1 mm^3^; (2) 170 slices with slice thickness of 1 mm^3^; (2) repetition time of 7.7 ms; (3) echo time of 3.5 ms; (4) bandwidth of 2.26 kHZ; (5) flip angle of 8 degrees; (6) field of view (FOV) of 256 × 200 × 170 mm; and (7) acquisition matrix size of 256 × 200.

The task-based functional MRI (fMRI) protocol obtained images using transverse echo-planar imaging (EPI) images in-plane with the AC-PC line. These were acquired using a gradient-echo pulse sequence and sequential slice acquisition (voxel dimension of 3.0 × 3.0 × 3.0 mm (i.e., 3 mm^3^); repetition time of 2000 ms, echo time of 30 ms, flip angle = 90°, 36 contiguous slices at 3 mm slice thickness, in-plane resolution of 80 × 80 pixels reconstructed in a field of view of 240 × 240 × 143 mm). Each functional run began with four scans during which no data were acquired to allow for steady state tissue magnetization. A total of 100 EPI volumes were collected in each functional run.

**Figure 2:**
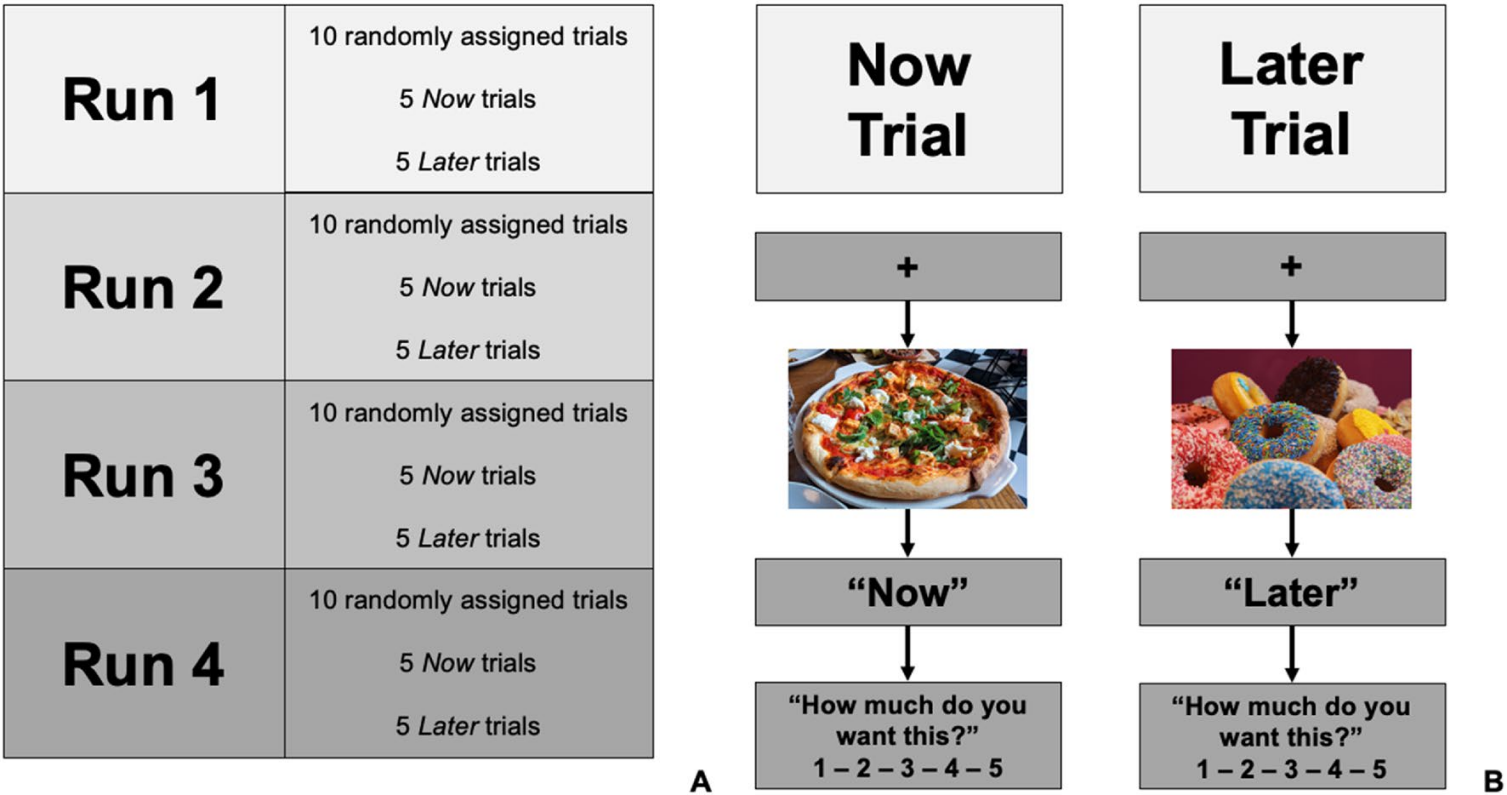
Illustration of the Now/Later task performed during functional magnetic resonance imaging (fMRI). (**A**) Illustration of run and block order for the *Now* and *Later* conditions. Each run consisted of 10 randomly ordered Now and Later tasks (5 Now/5 Later). Participants completed a total of 40 trials (20 Now; 20 Later). (**B**) Description of Now/Later task. Each trial began with a picture of a different food. For Now trials, participants were asked to think about how eating the pictured food would make them feel immediately. For Later trials, participants were asked to think about the long-term consequences of eating the pictured food. Participants were then asked to report how much they wanted the food.

During scanning, participants completed a version of the Now/Later delayed discounting executive control task (Fig. 2)^43^. Health behaviour choices are dependent upon executive control^22^. Indeed, health behaviours which are associated with executive control include: fruit and vegetable consumption, medication adherence, illicit drug use and alcohol consumption, and physical activity, among others^44^. The Now/Later task examines neural activation during delayed discounting, a component of executive control, by modeling cognitive regulation of food cravings during immediate (i.e., “Now”) versus delayed rewards (“Later”). Importantly, the task has been associated with numerous health behaviours, such as cigarette smoking, alcohol use, and food intake^43,45,46^. Participants completed four runs, with each run consisting of five randomly ordered *Now* trials and five randomly ordered *Later* trials For both *Now* and *Later* trials, participants were first shown a photographic image of a craving inducing food for ~ 8 s^45^. During *Now* trials, participants were instructed to consider the immediate consequences of consuming the pictured food (e.g., food taste or enjoyment.). For *Later* trials, participants were instructed to consider the long-term consequences of repeatedly consuming the food. Participants then rated their craving for the food using a 5-point Likert scale, during which time all functional images were obtained. Participants underwent 40 different trials with a pseudorandomized presentation order and intertrial intervals jittered ~ 3 s. The stimuli procedure and behavioural data were collected using the E-prime software system (Edition 2.0; Psychology Software Tools Inc., Sharpsburg, NC, USA).

Functional MRI analysis was performed using FSL (FMRIB’s Software Library, version 6.0.5.1; FMRIB Analysis Group, Oxford University, Oxford, UK). Task behavioural data underwent a quality control evaluation and initial pre-processing. Three participants were excluded due to corrupted stimulus timing files during data collection. Another participant was excluded due to errors in the design matrix during first-level processing. Thus, 26 participants were included in our analyses (I-INT = 13; D-INT = 13) and responded to 94% of all trials (94% of *Now* trials; 94% of *Later* trials).

All trials from the *Now* and *Later* tasks were analyzed. T_1_-weighted and functional images were inspected for excessive motion. Brain extractions of T_1_-weighted images were conducted using optiBET^47^. Preprocessed functional data from each subject were co-registered to subject’s high-resolution T_1_-weighted images and the Montreal Neurological Institute (MNI) standard space using FLIRT^48^. We applied motion correction using MCFLIRT^48^, spatial smoothing using a Guassian kernel of full-width half maximum at 6.0 mm, and high-pass temporal filtering (Gaussian-weighted least-squares straight line fitting, with sigma = 100 s).

A general linear model (GLM) identified neural activation in relation to separate event types. Trial-by-trial event stimulus onset was convolved via double-gamma function prior to constructing subject-level contrasts for the *Later–Now* condition.

### Statistical analyses

Our analyses were conducted in R version 4.1.2 and FSL version 6.0.5.1. All analysis code and output are available in a Github repository (see link). Descriptive analyses were used to summarize baseline characteristics. All analyses were conducted using only the 26 participants with valid task-based fMRI data.

#### Group differences in MVPA and SB over 6 months

We first examined whether there were treatment group differences among participants who underwent the Now/Later task in MVPA or SB at 2, 4, or 6 months after accounting for baseline differences. Linear mixed models using restricted maximum likelihood were conducted to examine changes in MVPA and SB by treatment group over 6 months. Time representing follow-up assessment (i.e., 2 months, 4 months, or 6 months) was included as a categorical fixed effect in addition to group and group × time interaction. The intercept was specified as a random effect and each model controlled for baseline outcome score. Unequal variance was allowed across time and group. Estimated marginal means, within group differences from baseline, and between group differences (I-INT–D-INT) at 2 months, 4 months, and 6 months follow-up were calculated.

#### Individual changes in MVPA and SB over 6 months

Next, we examined whether there were individual changes in MVPA and SB over 6 months among participants who underwent the Now/Later task. We developed separate linear mixed models using restricted maximum likelihood to determine individual changes over time in MVPA and SB from baseline to 6 months (i.e., MVPA slope and SB slope). Time representing each assessment point (i.e., baseline, 2 months, 4 months, or 6 months) was included as a fixed effect and categorical variable. The intercept and slope were specified as random effects. Given that we were interested in individual changes in MVPA and SB over time, no other covariates were included in this model. Unequal variance was allowed across time, and individual MVPA and SB slopes were predicted for each participant. We then plotted individual changes in MVPA and SB across time; MVPA and SB slopes were then used in our MRI analyses.

#### Task-based fMRI analyses: second-level group analysis

Lastly, we examined whether neural activation during the Now/Later task was associated with either (1) baseline MVPA/SB or (2) changes in MVPA over 6 months. Higher-level group analyses were carried out using FLAME (i.e., FSL’s local analysis of mixed effects) modelling and estimation (https://fsl.fmrib.ox.ac.uk/fsl/fslwiki/FEAT/UserGuide)^49^. Four separate single group average analyses were conducted to examine neural activation from the *Later*–*Now* contrast associated with (1) baseline MVPA; (2) baseline SB; (3) MVPA slope; and (4) SB slope. Our analysis focused on the *Later–Now* contrast because we were interested in the neural correlates associated with greater executive control. Models of baseline MVPA and SB controlled for age, biological sex, educational attainment, and BMI. Treatment group and baseline outcome performance were added as additional covariates for models of MVPA and SB slope.

The neurocognitive system which underlies MVPA and SB regulation is not well understood. Hence, we examined both positive and negative neural activation patterns which were associated with MVPA and SB. Positive neural activation patterns are reflective of increased blood-oxygen level dependent (BOLD) signal activity (i.e., greater blood flow to the specified brain area during task performance), while negative neural activation patterns are reflective of decreased BOLD signal activity^50^. Importantly, activation patterns are context dependent such that BOLD signal can imply both positive relationships to a task (i.e., greater activation of a brain region during a task promotes better performance), or a negative relationship (i.e., greater activation of a brain region during a task occurs in order to compensate for functional deficiencies in other brain regions). Positive and negative neural activation patterns were examined using a cluster correction threshold of *Z* > 2.3 and a (corrected) cluster significance threshold of *p* < 0.05^51^. We conducted separate contrasts for MVPA and SB, in order to determine the independent associations between brain activation patterns during the task and MVPA or SB. As a sensitivity analysis, we included the KOOS Pain Subscale as an additional covariate^38^.

Significant clusters were identified using *atlasquery* as implemented in FSL, and visually inspected in FSLeyes atlas view based on the MNI coordinates of the peak activation for each cluster and cluster subregions. We used both the Harvard–Oxford Cortical Structural Atlas, and the Harvard–Oxford Subcortical Structural Atlas for cluster identification and inspection (https://fsl.fmrib.ox.ac.uk/fsl/fslwiki/Atlases).

### Ethical approval

The research protocol was approved by the University of British Columbia Clinical Research Ethics Board (Application number: H14-01762), and registered with ClinicalTrials.gov (NCT02315664).

## Results

Twenty-six participants were included in our analyses (I-INT = 13; D-INT = 13) and provided baseline MVPA and SB data. As indicated in Fig. 1, one participant in the D-INT dropped out at two months and one participant in the I-INT dropped out at four months.

### Participant characteristics

Baseline characteristics are in Table 1. Mean age was 60 years (SD = 8 years), and 81% of the sample was female. Participants spent an average of 73.18 min/day in MVPA (SD = 51.02 min/day), and 690.67 min/day in SB (SD = 125.61 min/day) at baseline.

**Table 1:** Baseline participant characteristics for the full sample from the randomized controlled trial (N = 61) and the sample which underwent the fMRI analysis (N = 26).

	Analyzed sample (N = 26)		Full sample (N = 61)	
	Immediate intervention (N = 13)	Delayed intervention (N = 13)	Immediate intervention (N = 30)	Delayed intervention (N = 31)
Age	59.31 (7.09)	59.69 (9.84)	61 (9)	63 (9)
Females n, %	10, 76.92%	11, 84.62%	22, 73.33%	28, 90.32%
Education n, %				
High school or less	2, 15.38%	2, 15.38%	5, 16.67%	6, 19.35%
Some college or university	4, 30.77%	1, 7.69%	10, 33.33%	9, 29.03%
University degree or higher	7, 53.85%	10, 76.92%	15, 50%	16, 51.61%
BMI (kg/m^2^)	29.35 (4.67)	30.06 (4.79)	29.16 (5.46)	29.24 (4.82)
Montreal cognitive assessment	27.69 (1.38)	27.00 (1.90)	27.27 (2.53)	26.24 (2.86)
MVPA (min/day)^a^	80.96 (50.21)	65.41 (52.64)	83.44 (60.80)	86.19 (86.19)
SB (min/day)^b^	657.53 (109.24)	723.81 (136.23)	681.96 (111.51)	703.05 (161.17)
Now task craving ratings^c^	2.49 (1.37)	2.09 (1.24)	–	–
Later task craving ratings^c^	2.14 (1.15)	2.15 (1.16)	–	–
KOOS^d^ Pain Subscale	55.77 (13.33)	67.31 (15.21)	59.76 (16.05)	62.90 (17.17)

^a^Moderate-to-vigorous physical activity.

^b^Sedentary behaviour.

^c^Average craving ratings from 5-point Likert scale entered across each trial. Ratings are on a 1–5 scale with higher scores reflecting greater craving for the food.

^d^Knee Injury and Osteoarthritis Outcome Score.

### Group differences in MVPA and SB over 6 months

**Figure 3:**
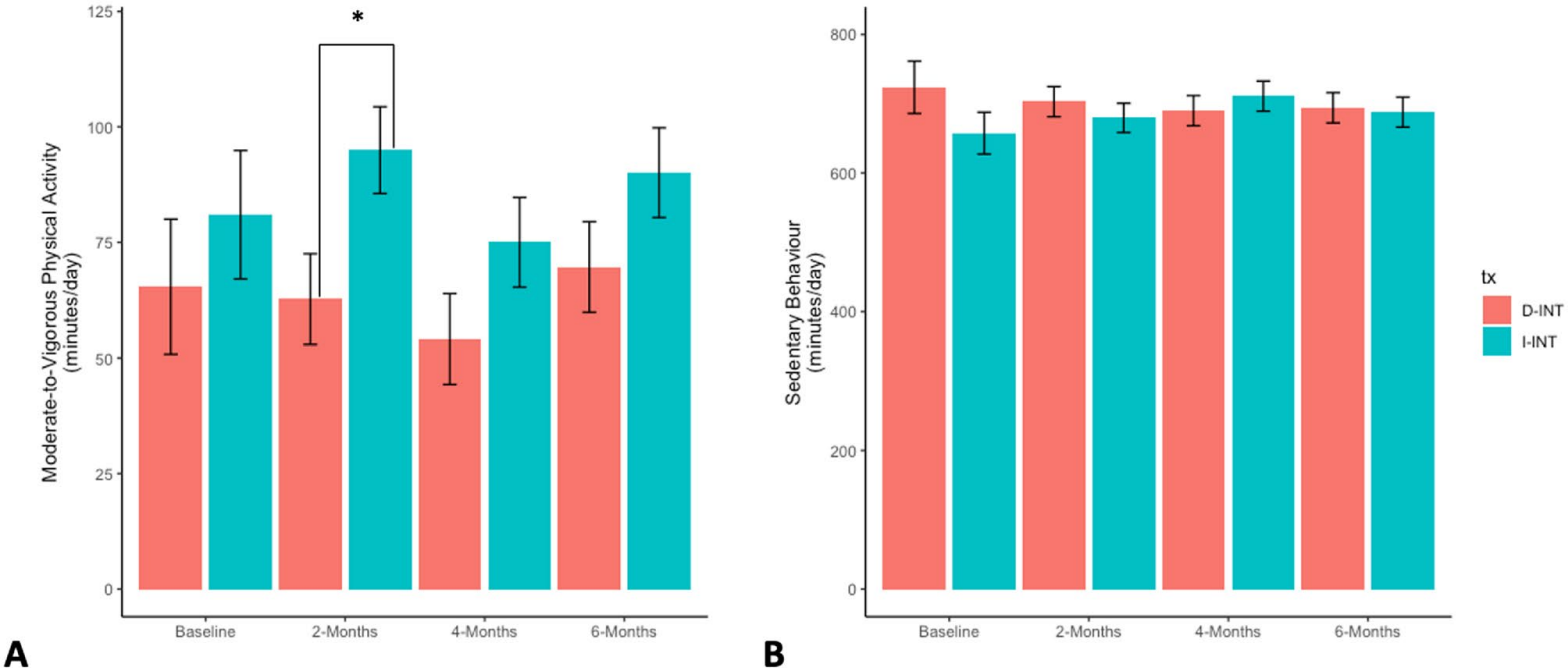
Estimated means and standard errors of moderate-to-vigorous physical activity (Panel A) and sedentary behaviour (Panel B) over the 6-month intervention. Significant differences between groups (*p* < 0.05) are denoted by *.

Estimated marginal means for MVPA and SB by treatment group and timepoint are described in Fig. 3. MVPA at two months was higher for the I-INT than the D-INT (estimated marginal mean difference: 32.26 min/day; 95% CI [4.96, 59.56]; *p* = 0.02). There were no significant differences between groups in SB at any timepoint. These findings echo the primary results of the study^33^.

### Individual changes in MVPA and SB over 6 months

**Figure 4:**
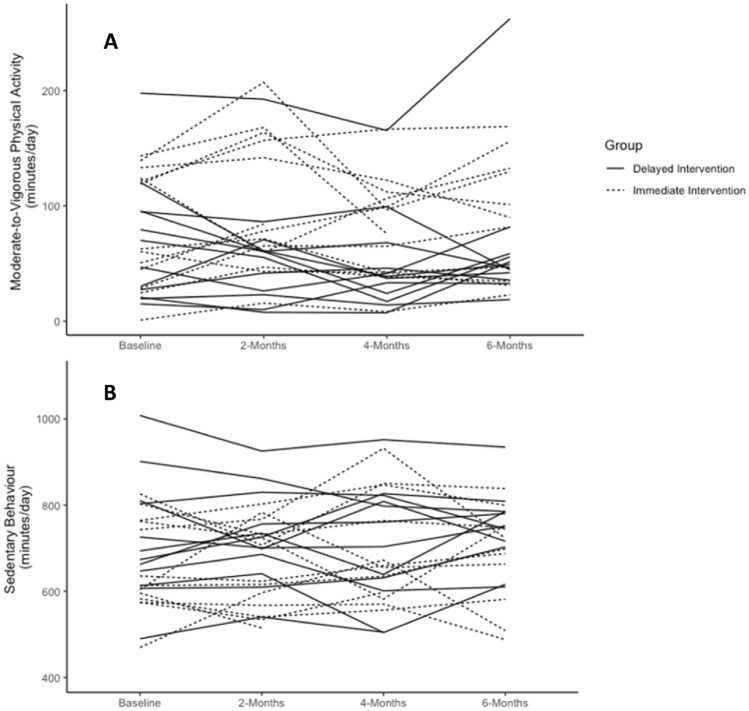
Individual changes in MVPA (**A**) and SB (**B**) over time stratified by group.

Figure 4 describes individual changes in MVPA and SB by timepoint. The MVPA slopes of participants in both groups ranged from a decline in MVPA of 11.47 min/day at each timepoint, to an increase in MVPA of 16.15 min/day at each timepoint. SB slope ranged from an increase in SB of 3.89 min/day at each timepoint to a decrease of 3.26 min/day. MVPA and SB slopes did not significantly differ by treatment group.

### Association of neural activation during Later–Now contrast with baseline MVPA or SB

**Figure 5:**
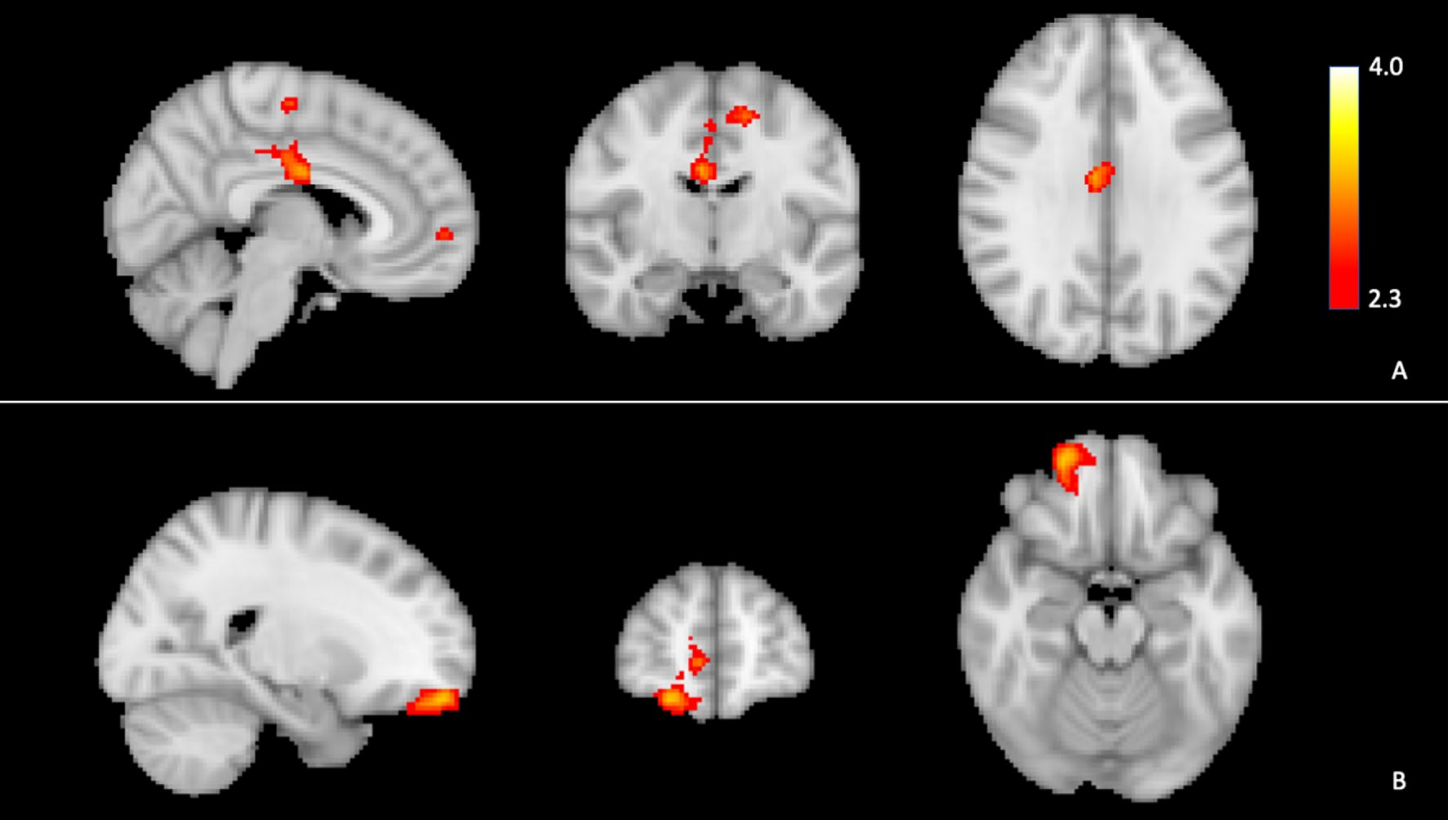
Associations of neural activity for Later – Now contrast with baseline moderate-to-vigorous physical activity (MVPA). Lower baseline MVPA was associated with greater neural activity in the right anterior cingulate gyrus (**A**) and right frontal pole (**B**). Neural activity signals are set to a threshold of *z* > 2.3 with brighter colours indicating a higher *z*. Model controlled for age, biological sex, educational attainment, and body mass index.

Cluster sizes and locations are in Table 2. Greater neural activation in the right anterior cingulate gyrus and the right frontal pole were associated with less MVPA at baseline (Fig. 5). There was no brain region wherein neural activation was associated with baseline SB.

**Table 2:** Cluster locations and sizes for models of now and later task.

Model	Location	Hemisphere	Voxels	Z-Max	Z-Max X (mm)	Z-Max Y (mm)	Z-Max Z (mm)	p-value
Baseline moderate-to-vigorous physical activity^a^			710					< 0.01
	(32%) Anterior cingulate gyrus	Right		3.4	6	− 10	30	
	(55%) Precentral gyrus	Right		3.33	2	− 16	60	
	(6%) Posterior cingulate gyrus	Right		3.20	14	− 22	34	
	(46%) Posterior cingulate gyrus	Right		3.20	4	− 16	36	
	(59%) Precentral gyrus	Right		3.20	2	− 18	54	
	(93%) Cerebral white matter	Left		3.11	− 12	− 12	58	
			500					0.04
	(63%) Frontal pole	Right		3.63	22	56	− 18	
	(44%) Frontal pole	Right		3.14	8	56	− 2	
	(97%) Cerebral white matter	Right		3.08	16	46	− 12	
	(65%) Frontal pole	Right		2.68	16	44	− 24	
	(24%) Paracingulate gyrus	Right		2.58	12	54	8	
	(98%) Cerebral white matter	Right		2.55	16	54	− 6	
Moderate-to-vigorous physical activity slope^b^			540					0.02
	(68%) Precuneus cortex	Right		3.57	8	− 60	52	
	(57%) Precuneus cortex	Left		3.38	0	− 54	52	
	(75%) Precuneus cortex	Left		3.02	− 8	− 50	44	
	(61%) Precuneus cortex	Left		3.00	− 6	− 66	52	
	(68%) Precuneus cortex	Right		2.97	8	− 48	48	
	(90%) Precuneus cortex	Right		2.97	4	− 48	48	

p-values represent significant clusters associated with either baseline MVPA or changes in MVPA over time irrespective of treatment group.

^a^Model controlled for age, biological sex, educational attainment, and body mass index (BMI).

^b^Model controlled for age, biological sex, educational attainment, BMI, treatment group, and baseline moderate-to-vigorous physical activity (MVPA).

### Association of neural activation during Later–Now contrast with MVPA or SB slopes

**Figure 6:**
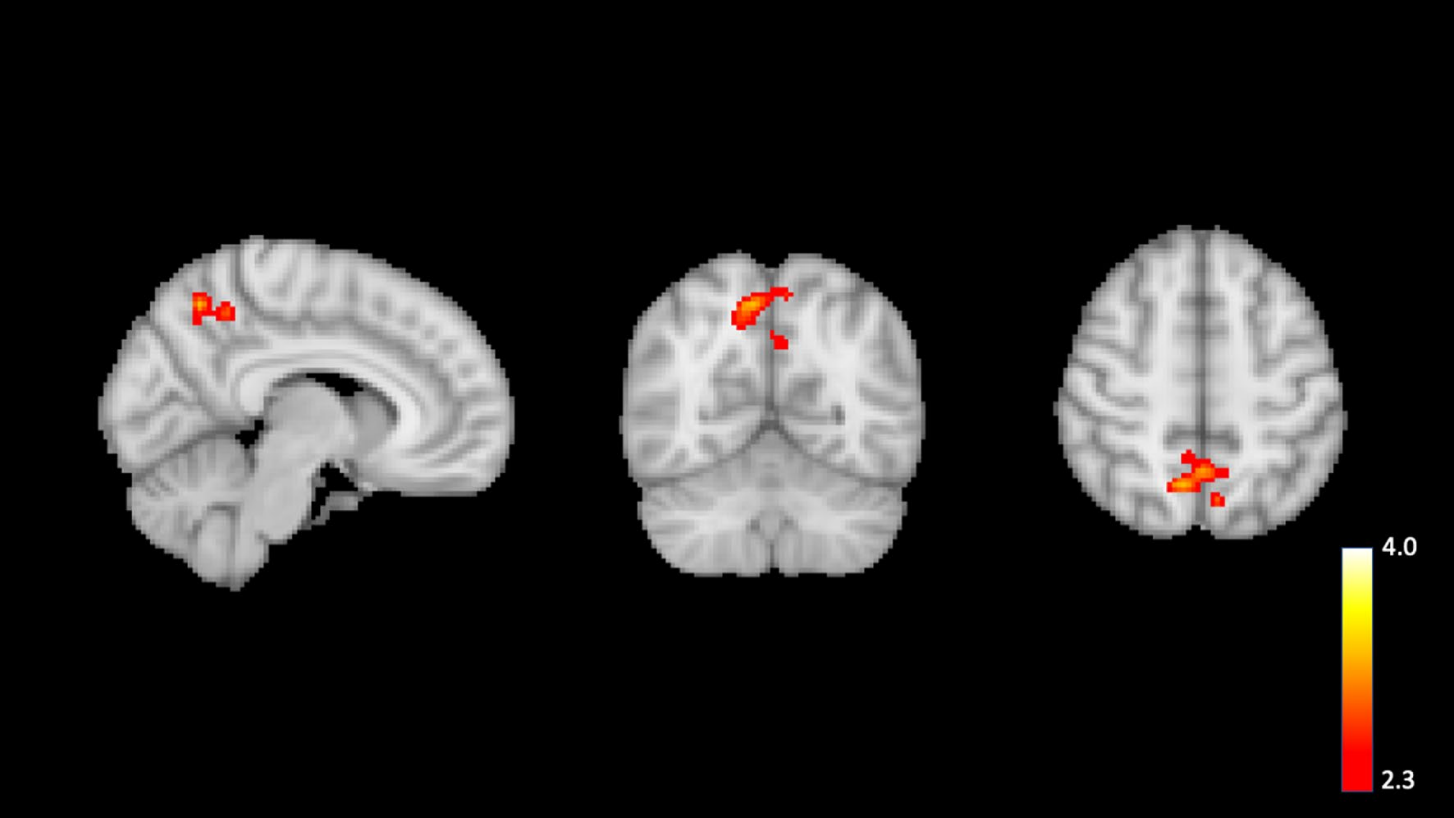
Associations of neural activity for Later–Now contrast with moderate-to-vigorous physical activity (MVPA) slope. Higher MVPA slope was associated with greater neural activity in the right precuneus cortex. Neural activity signals are set to a threshold of *z* > 2.3 with brighter colours indicating a higher *z*. Model controlled for age, biological sex, educational attainment, body mass index, treatment group, and baseline MVPA.

Greater neural activation in the right precuneus cortex at baseline was associated with greater increases in MVPA over time (Fig. 6). There was no brain region wherein neural activation at baseline was associated with SB changes over time.

### Sensitivity analysis—KOOS pain subscale as a covariate

In our cross-sectional analyses, greater neural activation in the right anterior cingulate gyrus was associated with less MVPA at baseline (Supplementary Materials S1). In our longitudinal analyses, greater neural activation in the right precuneus cortex and the left crus I of the cerebellum were associated with increases in MVPA over time (Supplementary Materials S2). There were not any brain regions which were associated with SB at baseline or with changes over time in SB.

## Discussion

Our results suggest a complex neurocognitive system is associated with MVPA. However, no neural correlates of executive control were associated with SB, either at baseline or over the 6-month study.

Multiple brain regions subserve the association between executive control and MVPA. At baseline, greater activation of the right anterior cingulate gyrus and the frontal pole were associated with less MVPA. The anterior cingulate and the frontal pole are associated with reward-guided decision making, whereby the anterior cingulate associates actions with rewards, while the frontal pole is involved in behavioural decision-making for future rewards^52^. MVPA does not typically provide immediate and tangible rewards in the modern world^53^. Indeed, engaging in MVPA may even be unpleasant for those who are already inactive, and thus it is plausible the anterior cingulate and frontal pole may be hyperactive during inhibition for people with low MVPA. Alternatively, adults with low MVPA might find engaging in inhibitory thoughts to be an aversive experience altogether, whereby neural activation would be higher among people with lower MVPA.

Notably, the precuneus predicted MVPA changes over the 6-month study period. Using a similar paradigm to our own, Striepens and colleagues^46^ determined that an intervention to increase executive control was associated with increased activation of the precuneus during the Now/Later task. Brody and colleagues determined that resisting cravings during a cigarette cue exposure involves the activation of the precuneus in cigarette smokers^54^. Collectively, these data suggest the importance of the precuneus in the self-regulation of multiple health behaviours, including MVPA.

Our findings thus suggest that MVPA is regulated by multiple overlapping networks of brain regions^55^. The anterior cingulate, frontal pole, and precuneus are each associated with several overlapping brain networks, including: the default mode network^56^, fronto-parietal network^57^, and cingulo-opercular network^58^. In particular, the fronto-parietal and cingulo-opercular networks display complex neural activity linked to executive control^59,60^. It is possible that increased neural activation of these regions may reflect changes in inter-network coupling (i.e., functional connectivity) associated with executive control. Assessing functional connectivity and neural flexibility associated with MVPA using the current task-based fMRI paradigm is beyond the scope of our study, and thus future research examining neural activity associated with MVPA should be conducted using paradigms designed to examine functional connectivity (e.g., resting state fMRI) in conjunction with task-based paradigms like ours.

While our results suggest that complex neural activity during executive control predicts adult MVPA, we did not find evidence of a neurocognitive system which predicted SB. These results are somewhat in contrast to the recent work of Morris and colleagues^32^, wherein the authors determined that functional connectivity between the (1) anterior cingulate and supplementary motor area; and (2) anterior insula and temporoparietal/temporooccipital junction predicted increases in SB over the course of a 6-month walking intervention. One plausible explanation for our finding is that the majority of our participants were highly sedentary and their SB did not change substantially over the course of the intervention—suggesting that a strong neurocognitive executive control system does not exist for SB. This seems especially plausible when one considers humans may have evolved to engage in SB whenever the opportunity arose^53^.

Our results also appear to support current hypotheses about the neurocognitive system (or lack thereof) which controls SB. Speakman hypothesized that SB appears to be an evolutionary adaptation which is “hard-wired” into our biology^20^. Cheval and Boisgontier postulated the theory of effort minimization for the prediction of physical activity and SB, wherein there is an automatic attraction towards minimizing effort and energy expenditure^19^. Our results thus provide some preliminary evidence which supports these hypotheses.

### Strengths, limitations, and future research

Our study has several strengths, including objective assessment of MVPA and SB, and cross-sectional and longitudinal analyses. One major limitation is that we did not conduct an a priori power calculation for this analysis, although we did aim to reduce Type I error by using a cluster correction threshold of Z > 2.3 and a corrected cluster significance threshold of *p* < 0.05.

Adults in this study were highly sedentary at study baseline and SB did not significantly change over time, which may potentially explain the lack of associations between SB and the fMRI task. Participant MVPA levels were also very high, with a mean MVPA at baseline of > 60 min/day; by comparison, average MVPA of adults 40+ years is < 30 min/day of MVPA^61^. Potentially this is because we did not exclude participants currently engaging in MVPA on a regular basis. Given that both MVPA and SB were both fairly high, but only MVPA showed a relationship with neural activation, our results should be treated with caution since it is plausible that the task performed did not appropriately capture executive control associated with SB. However, it is worth noting that the association between SB and executive function appears to be substantially weaker than the association between MVPA and executive function^25,26^.

All participants also had knee osteoarthritis; however, osteoarthritis is a common condition in later life wherein 9% of all men and 18% of all women over 60 years of age have osteoarthritis^62^. Our sample size was also small and predominantly female. We did not measure motivation or intent to increase MVPA or reduce SB. We did not control for seasonal effects in MVPA, nor did we account for differences in occupation which may have affected SB levels.

Task-based fMRI data was only collected at baseline. The Now/Later task which we employed used food cravings as a means of examining executive control. Although we used the same paradigm, images, and protocol as Kober and colleagues^43^, the same images were not repeated in both the Now and the Later trials such that it is plausible that activation patterns may have been dependent upon (1) the food image shown and; (2) the participant’s interest in that food. In addition, evidence of validity for this task as a measure of self-regulation is limited, even though the task is widely used as a means of quantifying neural activation during self-regulation^43,45,46^. Conceptually, this task has face validity as a means of activating the executive network during delayed discounting (i.e., thinking about the long-term negative consequences of eating a food versus the immediate consequences) which can be used as a proxy for examining self-regulation, but validation and confirmation studies are needed. At the present time, it is difficult for us to directly link this task with other executive control paradigms (e.g., Go-NoGo or Flanker task); however, the neural activation patterns associated with the Now/Later task involve the executive network, and thus have similar activation patterns to other executive control paradigms^63^.

While executive control in this task has been linked to other behaviours beyond food intake^43,45,46^, the neuralcorrelates of executive control which are associated with MVPA and SB may best be measured using a different paradigm. Importantly, there were no significant effects of the intervention on SB, and SB did not significantly change over time for either treatment group. Our findings suggesting that there is no neurocognitive system associated with SB should thus be treated with caution, and larger samples are needed to support our findings.

Given the exploratory nature of our study and the limited data on the neurocognitive system which regulates MVPA and SB, more research is needed in order to implement our findings into interventions. Future research should identify whether these neurocognitive systems can be intervened on in order to change MVPA and SB patterns. In addition, given that the Now and Later task may not be the best instrument for representing MVPA and SB self-regulation, there is a need to develop a new testing paradigm which represents MVPA and SB decision-making.

## Conclusion

Multiple brain regions underlying executive control were associated with MVPA, while no neural correlates were associated with SB. MVPA thus is associated with neurocognitive effort, while SB might be associated with the default behavioural pattern in adults. Future work examining neural activity associated with MVPA and SB should consider including both resting-state and task-based functional MRI approaches.

### Supplementary Information

Supplementary Information.

## Data Availability

Data are available upon reasonable request to TLA (teresa.ambrose@ubc.ca). All analysis code and output are available in a Github repository (https://github.com/ryanfalck/Now_and_Later).
