# Supplementary material for: Cross-sectional and longitudinal neural predictors of physical activity and sedentary behaviour from a 6-month randomized controlled trial
Source: Sci Rep. 2024 Jan 9;14:919. doi: 10.1038/s41598-023-48715-z (PMC10776740; doi:10.1038/s41598-023-48715-z)

Supplementary Materials S1. Cluster locations and sizes for sensitivity analyses of the Now and Later task

Model	Location	Hemisphere	Voxels	Z-Max	Z-Max X (mm)	Z-Max Y (mm)	Z-Max Z (mm)	p-value
Baseline Moderate-to-Vigorous Physical Activity[1]			914					<0.01
	(32%) Anterior Cingulate Gyrus	Right		3.38	6	-10	30	
	(55%) Precentral Gyrus	Right		3.27	2	-16	60	
	(54%) Precentral Gyrus	Right		3.19	2	-16	54	
	(95%) Cerebral White Matter	Right		3.19	16	-22	34	
	(56%) Precentral Gyrus	Right		3.14	2	-20	72	
	(97%) Cerebral White Matter	Left		3.13	-16	-16	36	
Moderate-to-Vigorous Physical Activity Slope[2]			469					0.04
	(74%) Left Crus I	Left		3.50	-36	-52	-36	
	(99%) Left Crus I	Left		3.24	-40	-56	-30	
	(92%) Left Crus II	Left		3.03	-40	-62	-44	
	(86%) Left Crus I	Left		2.93	-40	-58	-40	
	(19%) Left Cerebral Cortex	Left		2.92	-50	-72	-20	
	(72%) Left Crus I	Left		2.90	-44	-68	-24	
			742					<0.01
	(68%) Precuneous Cortex	Right		3.43	8	-60	52	
	(57%) Precuneous Cortex	Right		3.39	0	-54	52	
	(61%) Precuneous Cortex	Left		3.23	-6	-66	52	
	(75%) Precuneous Cortex	Left		3.00	-8	-50	44	
	(90%) Precuneous Cortex	Right		2.93	4	-48	48	
	(51%) Precuneous Cortex	Right		2.89	10	-50	46	

[1]Model controlled for age, biological sex, educational attainment, and body mass index (BMI)
[2]Model controlled for age, biological sex, educational attainment, BMI, treatment group, and baseline moderate-to-vigorous physical activity (MVPA)

Supplementary Materials S2. Sensitivity analyses of the associations of neural activity for Later – Now contrast with baseline moderate-to-vigorous physical activity (MVPA) and MVPA slope. Lower baseline MVPA was associated with greater neural activity in the right anterior cingulate gyrus (Panel A). Higher MVPA slope was associated with greater neural activity in the right precuneus cortex (Panel B) and the left crus I cerebellum (Panel C). Neural activity signals are set to a threshold of *z*>2.3 with brighter colours indicating a higher *z*. Model controlled for age, biological sex, educational attainment, body mass index, knee injury and osteoarthritis outcome severity (KOOS) score, and treatment group. Baseline MVPA was included as another covariate for our model MVPA slope.

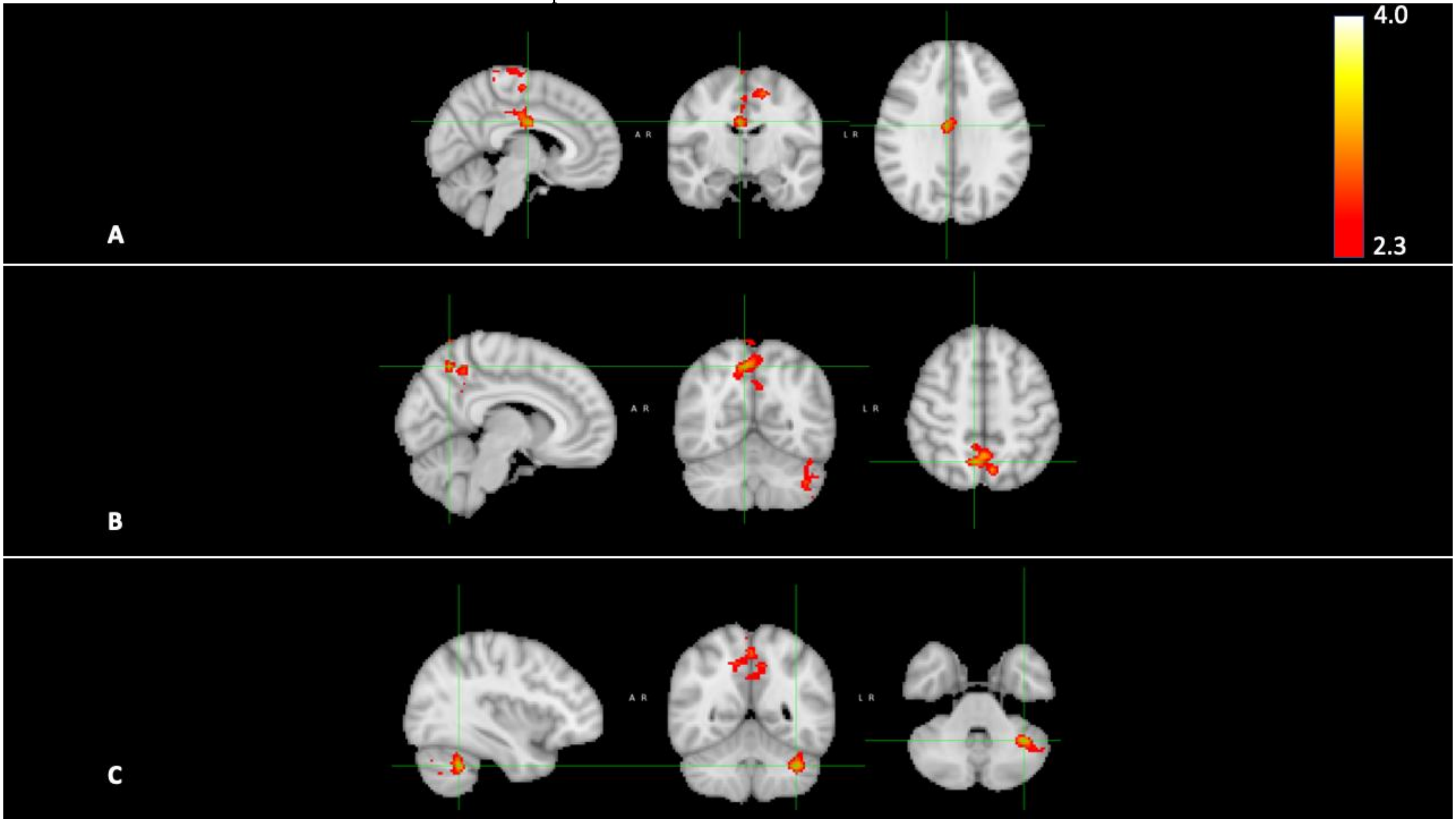

Supplement: Supplementary file 1 — Supplementary Information. [file 41598_2023_48715_MOESM1_ESM.pdf]
